# Supplementary material for: Beyond the pill: Understanding barriers and enablers to oral and long-acting injectable PrEP among women in sex work in Zambia
Source: PLOS Glob Public Health. 2025 Jun 4;5(6):e0004461. doi: 10.1371/journal.pgph.0004461 (PMC12136434; doi:10.1371/journal.pgph.0004461)
Supplement: S1 Table — (DOCX) [file pgph.0004461.s003.docx]

**S1 Table:** Potential PrEP Interventions for the Zambian Key Populations Investment Fund Informed by the COM-B Model

| **COM-B Component** | **Intervention** | **Description** |
| --- | --- | --- |
| Capability | Educational Workshops | - Conduct interactive workshops tailored to different literacy levels in languages spoken by WESW. - Use role-playing and Q&A sessions to educate about PrEP and its effectiveness even with alcohol use or during pregnancy. |
|  | Peer-led Education Programs | - Train peer navigators to provide accurate information and dispel myths about PrEP. - Create talking points or a brochure with popular misconceptions about PrEP. - Facilitate peer-led group discussions to share experiences and learn in a supportive environment. |
| Opportunity | Improving Access to Services | - Create a schedule to communicate PrEP refill dates via phone calls, and SMS reminders. |
|  | Convenient Service Delivery | - Expand PrEP refill depots to additional community-safe spaces like KI homes. - Conduct more regular outreach visits to venues such as brothels and bars to provide on-site PrEP services. |
| Motivation | Counseling and Support | - Offer personalized counseling to address individual concerns about PrEP. - Create support groups for WESW to discuss their experiences and challenges. |
|  | Positive Reinforcement | - Share success stories and testimonials from peers who have benefited from PrEP. - Fund more talktime for peer-navigators to call WESW initating on PrEP 2 weeks before their refill date. - Provide small incentives for follow-up appointment attendance for WESW newly intiating on PrEP. |
|  | Reducing Stigma | - Implement campaigns to reduce stigma associated with PrEP use, involving influential community members to promote positive attitudes towards oral and LAI-PrEP. |
| Combined Interventions Addressing Multiple COM-B components | Comprehensive Training Programs | - Train healthcare providers to deliver person-centered care. - Provide integrated health services combining PrEP with other sexual and reproductive health, substance use, and mental health services. |
|  | Enhanced Communication Strategies | - Implement regular follow-up via phone calls, SMS reminders, and home visits. - Create feedback mechanisms for WESW to voice concerns and suggestions. |
|  | Peer Support Networks | - Establish buddy systems for mutual support in persisting on PrEP. - Organize community events focused on health education and building a supportive network among WESW. |
