# Supplementary material for: Beyond the pill: Understanding barriers and enablers to oral and long-acting injectable PrEP among women in sex work in Zambia
Source: PLOS Glob Public Health. 2025 Jun 4;5(6):e0004461. doi: 10.1371/journal.pgph.0004461 (PMC12136434; doi:10.1371/journal.pgph.0004461)
Supplement: S1 Text — (DOCX) [file pgph.0004461.s004.docx]

# **S1 Text: In-Depth Interview Guide for Women Engaged in Sex Work or Peer-navigators**

**DEMOGRAPHICS**

1. What is your age?
2. What is your gender?
3. What is the highest level of education you have completed?
4. What is your marital status?

**ICEBREAKER**

1. What is your favorite food?
2. What is bringing you joy lately?

**PSYCHOSOCIAL STRESS**

**Q1**: Tell me your story, starting from how you started doing sex *work*?

**[PURPOSE OF QUESTION:** To allow the participant to become comfortable with the interviewer, and to tell their story in their own words.

**Q2:** What are some of the challenges of sex *work*?

**[PURPOSE OF QUESTION:** To allow the participant to discuss any stressors as a result of sex work**]**

**Q3:** How are these challenges different from those faced by men doing sex work?

**[PURPOSE OF QUESTION: T**o show vulnerabilities unique to women vs men involved in sex work?**]**

**Probe:**

- Stress related to setting where you work (eg: rest house, bottle store without much security)
- Family care for the children and stay where they work

**Q4:** Of the challenges you mentioned, which stress you a lot?

**[PURPOSE OF QUESTION:** To allow the participant to explain what they feel are the most important stressors in their life. This also provides context for how this person makes decisions around their health.**]**

**Q5**: What has made those stressors better?

**[PURPOSE OF QUESTION:** To find out about social capital and pathways of resiliency**]**

**Probe:**

- Support from other women engaged in sex work, for example a queen mother
- Peer-navigators
- Services at KPIF

**HISTORY OF HIV PREVENTION**

**Q6:** What are the different things you use to prevent HIV?

**[PURPOSE OF QUESTION:** To establish baseline knowledge of HIV prevention measures**]**

**Probes**:

- Understanding of available HIV prevention tools
- (ex: condoms, or PrEP, PostExposure Prophylaxis (PEP) especially for victims of violence and sexual abuse – Zambian National Guideline states it should be taken within 72 hours of the unwanted sexual encounter)

**Q7:** How do you go about getting these prevention services?

**[PURPOSE OF QUESTION:** To prompt the participant to think of places where they accessed HIV prevention services]

**Q8:** What has been your good or bad experiences in getting these prevention services?

**[PURPOSE OF QUESTION:** To prompt the participant to think of the quality of HIV prevention services they received**]**

**Q9:** Based on your experiences, how do you feel about the health care services that are available to you?

**[PURPOSE OF QUESTION:** To prompt the participant to think of instances that created distrust in health care services**]**

**Probes**:

- Being refused health services
- Uncomfortable revealing your identity to health providers

**Q10**. How did you happen to choose this space for your health services?

**[PURPOSE OF QUESTION:** To encourage the participant to think of what positive qualities prompted them to give the program a chance and access this space**]**

**Probes**:

- Social network referred you,
- Convenience - less wait time, drugs are stocked,
- friendly, non-stigmatizing, non-discriminating

**PrEP-SPECIFIC QUESTIONS**

**Note to Interviewer:** This study is recruiting women who were eligible to initiate on PrEP in the last 3 months and asking them to reflect on their experience.

**Q11.** Did you start taking PrEP?

**Q12.** You said you did not start PrEP, what factors stopped you from starting PrEP?

**[PURPOSE OF QUESTION: To understand barriers to initiating PrEP]**

**Probes**:

- - - Was not offered PrEP.
    - Did not receive enough information about it.

**Q13.** You said you did start PrEP, what made you decide to take PrEP?

**[PURPOSE OF QUESTION: To understand motivating factors for decision to initiate PrEP]**

**Probes**:

- wanted to protect myself,
- protect my partner,
- stay healthy,
- continue care-taking for those who depend on me

**Q14.** What helped you to start taking PrEP?

**[PURPOSE OF QUESTION: To identify what the enabling factors to initiating PrEP]**

**Probe**:

- peer network,
- support from health workers,
- convenience of site,
- site had drugs in stock

**Q15.** The most common ways of getting infected with HIV are sex without condoms, injecting drugs. Tell me about your risk of HIV exposure in the last 3 months. Were they what you envisioned when you started PrEP?

**[PURPOSE OF QUESTION: To understand whether PrEP use was aligned with perception of risk]**

**Q16.** If you are still taking PrEP, what makes you continue taking it?

**[PURPOSE OF QUESTION: To identify enablers to continuing to persist on PrEP over time]**

**Q17:** If you are not still taking PrEP, what made you stop taking it?

**[PURPOSE OF QUESTION: To identify the barriers to persisting on PrEP over time]**

**Probe**:

- Stigma

**INJECTABLE PREP**

**INSTRUCTION:**

Give patient education on oral PrEP vs long acting injectable PrEP**.**

Injectable PrEP is very effective at protecting people from HIV and is for adults and adolescents who are at risk of getting HIV through sex. It is a jab given every 2 months instead of taking pills everyday. The medicine in the jab is in your blood for 2 months and prevents HIV infection. The medicine is the same type of medicine as in the pills. Injectable PrEP is being used in other countries, and will be available in Zambia in the next year.

**Q18a.** What do you think are the advantages of taking injectable PrEP for women who exchange sex for money?

**Q18b.** What are the disadvantages?

**[PURPOSE OF QUESTION: To gauge advantages and disadvantages of injectable PrEP which will be newly available in Zambia.]**

**Q19a**. What are some reasons why women who exchange sex for money would like the jab every 2 months to prevent HIV?

**Q19b**. What are some reasons why they would not like the jab every 2 months?

**[PURPOSE OF QUESTION: To gauge acceptability of injectable PrEP which will be newly available in Zambia.]**

**SERVICE IMPROVEMENT**

**Q20a.** What factors may lead you to trust any clinic or health care providers less?

**Q20b.** What made you trust the clinic or health providers more?

**[PURPOSE OF QUESTION: To understand what makes services trustworthy to the participant.]**

**Probe**:

· Closed vs Open spaces

· Women vs men

· Privacy / confidentiality

· Having a peer from the community assist me

· Having someone who has had experience in sex work

**Q21**. What should we change in this space to make it more supportive for you to get HIV prevention services? How will this help you?

**[PURPOSE OF QUESTION: To understand specific services women would want from a ‘safe space’ and understand how they perceive the provision of those services would affect their health]**

**Probes:**

· Getting a one-stop shop of services (family planning, STIs, substance use, mental health)

· Information on economic / training opportunities / women’s empowerment

· Food packages/ nutritional assistance

· Transportation assistance to make it to health appointments

**Q22**. Who would you want supporting you to stay on PrEP when you feel you need to be on PrEP?

**[PURPOSE OF QUESTION: To understand who is best to deliver prevention services ]**

**STIGMA-SPECIFIC QUESTIONS**

**Q23**: Earlier you referred to stigma from XX and XX. What are ways we can reduce stigma?

**[PURPOSE OF QUESTION: To identify stigma reduction interventions]**

**Probes**:

- Stigma training programs for health care workers
- Is there anything we can do for you?

**Q24**: Is there anyone within your work network who attitudes we could change if there was a program to reduce stigma?

**[PURPOSE OF QUESTION: To identify people who would benefit from stigma reduction interventions. To assess efificacy, is there anything we could do to change this? ]**

*Please read verbatim:*

[“Thank you for taking the time to share your story with me today. Do you have any questions or things you would like to talk about before we end the interview?”]
