# Supplementary material for: Beyond the pill: Understanding barriers and enablers to oral and long-acting injectable PrEP among women in sex work in Zambia
Source: PLOS Glob Public Health. 2025 Jun 4;5(6):e0004461. doi: 10.1371/journal.pgph.0004461 (PMC12136434; doi:10.1371/journal.pgph.0004461)
Supplement: S2 Checklist — (DOCX) [file pgph.0004461.s005.docx]

Inclusivity in global research

PLOS’ policy on inclusivity in global research aims to improve transparency in the reporting of research performed outside of researchers’ own country or community and ensures that PLOS publications reporting global research adhere to high standards for research ethics and authorship. Authors of relevant research articles may be asked to complete the questionnaire below, which outlines ethical, cultural, and scientific considerations specific to inclusivity in global research. This questionnaire may be requested when researchers have travelled to a different country to conduct research, if research uses samples collected in another country, research with Indigenous populations or their lands, or if research is on cultural artefacts. Researchers travelling to another country solely to use laboratory equipment will not normally be required to complete the questionnaire. However, the questionnaire can be requested at the journal’s discretion for any submission – if you have been requested to complete this questionnaire by the PLOS journal you submitted to, please do so.

Please complete the questionnaire below and include this as a Supporting Information file with your manuscript. Note that if your paper is accepted for publication, this checklist will be published with your article in the supporting information files. Please ensure that you reference the checklist in the main body of your manuscript. We suggest adding a subsection ‘Inclusivity in global research’ to your Methods section and adding the following sentence: “Additional information regarding the ethical, cultural, and scientific considerations specific to inclusivity in global research is included in the Supporting Information (SX Checklist)”

The questions have been designed to be applicable to a wide range of study types, and there are subsections for both human subjects research and non-human subjects research. If any of the questions are not relevant to your research please mark them as “N/A” as appropriate.

**Ethical considerations, permits and authorship**

*This section is applicable to all research types.*

Provide details as to who granted permissions and/or consent for the study to take place in the Methods section of your manuscript. This should include the names of **all** ethics boards, governmental organizations, community leaders or other bodies that provided approval for the study. If individuals provided approval refer to these people by their role or title but do not list their name(s).

Reported on page number: pg 11: This study was approved by the University of Zambia Biomedical Research Ethics Committee (UNZABREC; protocol 3650-2023), dated 21/06/2023 and the University of North Carolina (UNC) Biomedical Institutional Review Board (IRB) (22-3147), dated 11/07/2023.

If there were any deviations from the study protocol after approval was obtained please provide details of these changes in the Methods section of your manuscript.
Did this study involve local collaborators that are residents of the country where the research was conducted or members of the community studied? If you do not have any authors from said communities, please provide an explanation for this below.

Reported on page number:

NA- no deviations.

Everyone listed as an author should meet PLOS’ criteria for authorship and all individuals who meet these criteria should be included in the author byline, rather than the acknowledgements. For further information please see the journal’s Authorship Policy.

Yes there are several local collaborators who also serve on the community advisory board: Ruth Zyambo, Martin Zimba who are also key sex work community members and leaders of civil society organizations who co-created this study protocol.

Citation: Kumar R, Rao D, Sharma A, et alMixed-methods protocol for the WiSSPr study: Women in Sex work, Stigma and psychosocial barriers to Pre-exposure prophylaxis in ZambiaBMJ Open 2024;14:e080218. doi: 10.1136/bmjopen-2023-080218

**Human subjects research (e.g. health research, medical research, cross-cultural psychology)**

Did you obtain written informed consent from a representative of the local community or region before the research took place? How did you establish who speaks for the community? Details of written informed consent obtained from study participants should be reported separately in the Methods section of your manuscript.

The Zambian National Health Research Authority as well a the Lusaka Provincial and Lusaka District Ministry of Health offices all gave their written permission for this research study to take place. In addition, the Lusaka District Ministry of Health specialist for key population, along with key members of civil society organizations as well as the sex work community provided oversight for this study through a community advisory board. The details of this board and the co-creation of the study protocol, informed consent, and survey instruments is details in the protocol.

Citation: Kumar R, Rao D, Sharma A, et alMixed-methods protocol for the WiSSPr study: Women in Sex work, Stigma and psychosocial barriers to Pre-exposure prophylaxis in ZambiaBMJ Open 2024;14:e080218. doi: 10.1136/bmjopen-2023-080218

How did members of the local community provide input on the aims of the research investigation, its methodology, and its anticipated outcome(s)?

When engaging with the local community, how did you ensure that the informed consent documents and other materials could be understood by local stakeholders?

Will the findings of the research be made available in an understandable format to stakeholders in the community where the study was conducted (e.g. via a presentation, summary report, copies of publications, etc.)? Please provide details of how this will be achieved.

The informed consent was translated into multiple local languages (Nyanja and Bemba), and an additional informed consent form quiz was administered to really ensure that participants understood the risks of being in this study. All of these were cleared by the community advisory board and detailed in the original study protocol (citation above).

The interview guides were developed with input from a community advisory board, and piloted with board members and senior peer-navigators. The interviews were conducted with caution in a private room at the community wellness drop-in centre, or in the private homes of senior members of the sex work community. The well-being of our pariticipants was the central consideration of the study, which was considered as a driver of the study design and the reasons for the involvement of community advisory board. Civil society organization staff and key informants were not present in the interview room, but they did monitor the emotional reactions of participants after the interview and provided psychological support to the participants if needed. No repeat interviews were done.

Our positionality as researchers, including our socio-cultural backgrounds, education, and professional roles, influenced both the design of the interview questions and our interpretation of the results. We acknowledge that these factors may have shaped the framing of our questions and our understanding of participants' responses, and we sought to address this by incorporating feedback from a community advisory board and peer navigators throughout the research process.

Final research findings were disseminated to the community hubs where recruitment took place and validated by community members with the support of the advisory board and key informants who were involved every step of the research process.

**Non-human subjects research using specimens/ animals collected as part of the study, or those housed in archival collections. Examples include archaeology, paleontology, botany and zoology.**

Did the permission you obtained from a local authority to perform the study include an agreement on access to outputs and benefit sharing? This may include procedures to enable fair distribution of the benefits and resources arising from the research performed. Please include any details of Prior Informed Consent and Benefit Sharing Agreements obtained. These may be required by field-specific regulations, for example the Convention on Biological Diversity (CBD) and the associated Nagoya Protocol.

NA

If the material used in your study was imported, please A) provide the year it was imported and B) indicate whether permits were obtained to import/export the materials used, C) provide details of any permits obtained. If this information is not available, please indicate this.

NA

If you used archival specimens, please state how the material used in your study was acquired by the institute it is held in and provide details of any permits obtained for the original excavations/ sample collection. If this information is not available, please indicate this.

NA

How was the potential cultural significance of the materials collected in your study to local communities considered in your research design? Were Indigenous peoples and/or local researchers and institutions involved with archaeological excavations / collection of specimens? If so, please provide a description of their involvement.

NA

If your manuscript includes photographs of human remains please indicate whether authors obtained permission from descendants or affiliated cultural communities to do so.

NA
