# Supplementary material for: Beyond the pill: Understanding barriers and enablers to oral and long-acting injectable PrEP among women in sex work in Zambia
Source: PLOS Glob Public Health. 2025 Jun 4;5(6):e0004461. doi: 10.1371/journal.pgph.0004461 (PMC12136434; doi:10.1371/journal.pgph.0004461)
Supplement: Attachment — Submitted filename: ResponsetoReviewers.docx [file pgph.0004461.s007.docx]

**Response to Reviewers**

PGPH-D-24-02335
Beyond the Pill: Understanding Barriers and Enablers to Oral and Long-Acting Injectable PrEP Among Women in Sex Work in Zambia.
PLOS Global Public Health

Journal Requirements:

1. Please include a complete copy of PLOS’ questionnaire on inclusivity in global research in your revised manuscript. Our policy for research in this area aims to improve transparency in the reporting of research performed outside of researchers’ own country or community. The policy applies to researchers who have travelled to a different country to conduct research, research with Indigenous populations or their lands, and research on cultural artefacts. The questionnaire can also be requested at the journal’s discretion for any other submissions, even if these conditions are not met.  Please find more information on the policy and a link to download a blank copy of the questionnaire here: <https://journals.plos.org/globalpublichealth/s/best-practices-in-research-reporting>. Please upload a completed version of your questionnaire as Supporting Information when you resubmit your manuscript.

2. Please ensure that the Title in your manuscript file and the Title provided in your online submission form are the same.

We have cross-checked the title in the submission form and file and changed the file to be “Beyond the Pill: Understanding Barriers and Enablers to Oral and Long-Acting Injectable PrEP **Among** Women in Sex Work in Zambia.”

3. We have amended your Competing Interest statement to comply with journal style. We kindly ask that you double check the statement and let us know if anything is incorrect.

We downloaded the current manuscript that was in the system but did not see any modifications to the Competing Interest statement. We can review it with the proof before publication.

4. In the online submission form, you indicated that "Due to the sensitive nature of the research involving a small sample of marginalized women and the need to protect participant confidentiality, the interview data are not publicly available. Access to de-identified transcripts or other supporting information may be provided upon reasonable request, subject to ethical approvals and institutional review board guidelines".

All PLOS journals now require all data underlying the findings described in their manuscript to be freely available to other researchers, either 
a. In a public repository, 
b. Within the manuscript itself, or 
c. Uploaded as supplementary information.

This policy applies to all data except where public deposition would breach compliance with the protocol approved by your research ethics board. If your data cannot be made publicly available for ethical or legal reasons (e.g., public availability would compromise patient privacy), please explain your reasons by return email and your exemption request will be escalated to the editor for approval. Your exemption request will be handled independently and will not hold up the peer review process, but will need to be resolved should your manuscript be accepted for publication. One of the Editorial team will then be in touch if there are any issues.

We have uploaded the data from the rapid qualitative review memos as a supplementary file and stripped all identifiers. This is due to the sensitive nature of the research involving a small sample of marginalized women and the need to protect participant confidentiality. It is possible to use contextual clues and information about the age and location of the interview participants to identify their real names and this would cause them serious harms in an environment where sex work and transgender identities are criminalized. Access to de-identified transcripts or other supporting information may be provided only upon reasonable request.

[Note: HTML markup is below. Please do not edit.]

Reviewers' comments:

Reviewer's Responses to Questions

**Comments to the Author**

1. Does this manuscript meet PLOS Global Public Health’s [publication criteria](https://track.editorialmanager.com/CL0/https:%2F%2Fjournals.plos.org%2Fglobalpublichealth%2Fs%2Fcriteria-for-publication/1/010f0193c06a0562-70d520ea-1c6f-44a4-9e09-5d26128b3d22-000000/Zzs7AM_ksFphgJuPbUJDjKNZhparJaW0G8K4V-X_68o=189)? Is the manuscript technically sound, and do the data support the conclusions? The manuscript must describe methodologically and ethically rigorous research with conclusions that are appropriately drawn based on the data presented.

Reviewer #1: Yes

Reviewer #2: Yes

2. Has the statistical analysis been performed appropriately and rigorously?

Reviewer #1: N/A

Reviewer #2: N/A

3. Have the authors made all data underlying the findings in their manuscript fully available (please refer to the Data Availability Statement at the start of the manuscript PDF file)?

The [PLOS Data policy](https://track.editorialmanager.com/CL0/https:%2F%2Fjournals.plos.org%2Fglobalpublichealth%2Fs%2Fdata-availability/1/010f0193c06a0562-70d520ea-1c6f-44a4-9e09-5d26128b3d22-000000/xUH9Kg0RcMeUh1HSp_DUy_hriJuehtW-uHIpDWU68Lc=189) requires authors to make all data underlying the findings described in their manuscript fully available without restriction, with rare exception. The data should be provided as part of the manuscript or its supporting information, or deposited to a public repository. For example, in addition to summary statistics, the data points behind means, medians and variance measures should be available. If there are restrictions on publicly sharing data—e.g. participant privacy or use of data from a third party—those must be specified.

Reviewer #1: Yes

Reviewer #2: Yes

4. Is the manuscript presented in an intelligible fashion and written in standard English?

PLOS Global Public Health does not copyedit accepted manuscripts, so the language in submitted articles must be clear, correct, and unambiguous. Any typographical or grammatical errors should be corrected at revision, so please note any specific errors here.

Reviewer #1: Yes

Reviewer #2: Yes

5. Review Comments to the Author

Please use the space provided to explain your answers to the questions above. You may also include additional comments for the author, including concerns about dual publication, research ethics, or publication ethics. (Please upload your review as an attachment if it exceeds 20,000 characters)

Reviewer #1: General:
Thank you for allowing me to review this interesting and well-written article on a very relevant, timely and important subject. I commend the authors for their thoughtful approach on conducting the research, which generated very insightful and nuanced insights, conveyed in a very structured and clear way. My comments are mainly minor, aiming to clarify and further improve the manuscript.

We appreciate your positive reception of this work.

Abstract:
1. Line 40 (conclusion): the abbreviation “KP” is used but has not been introduced earlier. I suggest to spell this out.

This has been changed to “WESW” which was previously defined.

Introduction:

2. The authors could consider giving some absolute numbers on women who sell sex affected by HIV on a global (and/or local) scale, to give an indication of the scope of the problem.

Thank you for the feedback. The vast majority of countries (168) still criminalize some aspect of sex work. Survey data on these populations is modeled and most studies prefer to eport HIV prevalence as a percentage rather than absolute numbers due to the challenges of obtaining reliable population estimates. However, global estimates suggest that women who sell sex are disproportionately affected by HIV, with prevalence rates ranging from 30% to 60% in some regions. In Zambia, studies indicate similarly high prevalence rates, emphasizing the urgency of targeted interventions.

**Citations**:

Stevens, O, Anderson, R, Stover, J, Teng, Y, Stannah, J, Silhol, R, Jones, H, Booton, RD, Martin-Hughes, R, Johnson, L, Maheu-Giroux, M, Mishra, S, Stone, J, Bershteyn, A, Kim, H-Y, Sabin, K, Mitchell, KM, Dimitrov, D, Baral, S, Donnell, D, Korenromp, E, Rice, B, Hargreaves, JR, Vickerman, P, Boily, M-C & Imai-Eaton, JW 2024, 'Comparison of empirically derived and model-based estimates of key population HIV incidence and the distribution of new infections by population group in sub-Saharan Africa', Journal of Acquired Immune Deficiency Syndromes, vol. 95, no. 1S, pp. e46-e58. <https://doi.org/10.1097/QAI.0000000000003321>

Shannon K, Crago AL, Baral SD, Bekker LG, Kerrigan D, Decker MR, et al. THE GLOBAL RESPONSE AND UNMET ACTIONS FOR HIV AND SEX WORKERS. Lancet. 2018 Aug 25;392(10148):698–710.

3. Line 74: spelling mistake “three times higher at 33.3%”.

We have changed this to ‘higher’.

4. Line 76: do you have a reference to support the statement that “failure to protect WESW will prolong the pandemic indefinitely at a huge cost” (e.g. is this based on modelling or personal projec	tions)?

We removed the word ‘indefinitely’ and added a citation .

Shannon K, Crago AL, Baral SD, Bekker LG, Kerrigan D, Decker MR, et al. THE GLOBAL RESPONSE AND UNMET ACTIONS FOR HIV AND SEX WORKERS. Lancet. 2018 Aug 25;392(10148):698–710.

5. Line 78: PrEP is not only recommended by WHO for key populations but all at increased risk of HIV.

Changed KP to ‘anyone.”

6. Line 83-92: I felt this paragraph could have benefited from some more interaction with the existing literature on (structural) barriers to PrEP. It would be good to include some references here.

We agree and thanks for pointing this out. We have added 4 citations here in the literature:

Ghayda RA, Hong SH, Yang JW, Jeong GH, Lee KH, Kronbichler A, et al. A Review of Pre-Exposure Prophylaxis Adherence among Female Sex Workers. Yonsei Med J. 2020 May;61(5):349–58.

6.	Cowan FM, Delany-Moretlwe S. Promise and pitfalls of pre-exposure prophylaxis for female sex workers. Curr Opin HIV AIDS. 2016 Jan;11(1):27–34.

7.	Syvertsen JL, Bazzi AMR, Scheibe A, Adebajo S, Strathdee SA, Wechsberg WM. The Promise and Peril of Pre-Exposure Prophylaxis (PrEP): Using Social Science to Inform PrEP Interventions among Female Sex Workers. Afr J Reprod Health. 2014 Sep;18(3 0 0):74–83.

8.	Shannon K, Strathdee SA, Goldenberg SM, Duff P, Mwangi P, Rusakova M, et al. Global epidemiology of HIV among female sex workers: influence of structural determinants. The Lancet. 2015 Jan 3;385(9962):55–71.

7. Line 93-96: the authors may want to consider giving some more detail on the geographic regions and populations among which the LAI-PrEP trials were conducted.

We have added the following information L96-100:

The LAI-PrEP trials were conducted among distinct populations in different geographic regions. The HPTN 084 trial was conducted in seven African countries among HIV-uninfected cisgender women, while the HPTN 083 trial enrolled cisgender men who have sex with men and transgender women who have sex with men across multiple global sites. Detailed findings are available in Delany-Moretlwe et al. (2022) and Landovitz et al. (2021).

8. Line 117: It was not clear to me what was meant by “reachable moments” – consider just talking about “reach”?

A "reachable moment" refers to a critical point in time when an individual is most open to receiving health interventions, support, or behavior change messaging. In the context of PrEP uptake, these moments may occur during routine healthcare visits, HIV testing, outreach programs, or after experiencing a significant life event (e.g., a recent HIV exposure scare or a peer’s HIV diagnosis). Recognizing and leveraging these moments can enhance the effectiveness of HIV prevention efforts by meeting individuals where they are most receptive.

We have clarified this in the text L120-123.

**Methods:**
9. Line 129: I was wondering whether the aim of the parental study could be mentioned. Reason being that I was interested to know whether there was quantitative data available on the uptake and continuation rates of PrEP among WESW in this context. This could be useful to contextualise some of the qualitative findings as well.

Yes there was quantitative data and this paper is being published separately. We have clarified in the text (L129-133) that: This study is part of a larger parent study that also includes quantitative data to measure the association between multiple stigmas and PrEP initiation and persistence among HIV-negative adult WESW[16]. The qualiative findings of this paper will complement and contextualise findings from the quantitative results[17,18].

10. Consider including a visual for the COM-B model?

We have included a visual and on L149 we have made reference to the COM-B model graphic which is from citation:

McDonagh, L.K., Saunders, J.M., Cassell, J. et al. Application of the COM-B model to barriers and facilitators to chlamydia testing in general practice for young people and primary care practitioners: a systematic review. Implementation Sci 13, 130 (2018). https://doi.org/10.1186/s13012-018-0821-y

11. Line 153: I would suggest to use “effectiveness” instead of “efficacy” when talking about real-world implementation of these interventions.

Good catch thanks for making this point. We have changed it on linke 165.

12. Participant selection: were participants sampled from the quantitative survey, or how was this done?

They were sampled from 300 women who were recruited to the quantiative survey. This is in the main protocol and cited in the paper.

13. Data analysis: It was not entirely clear to me whether the final analysis was based on the summary memos (step 1) or on the generated verbatim transcripts?

In addition, am I understanding it correctly that the codebook included the COM-B constructs as higher-level (deductive) categories, but that themes under these categories were still inductively delineated? If so, I would consider phrasing it like this (and not refer to the COM-B construct as “themes” to avoid confusion).

We have specifyed the final analysis is on the memos - see Line 260.

We also specify that this analysis of memos is avialable in the supplement - see line 266

We have specifyed that we identified higher-level deductive categories (line 262) and we double-checked and ensured that we have referred to the COM-B domains throughout this manuscript, and do not refer to the COM-B constructs as “themes.”

14. Nice to include a critical reflexivity statement!

We appreciate your positive comment!

Results:
15. Line 319-322: this difference in trust among participants in the program’s services is attributed (by the authors?) to time (“building trust takes time”). However, is this an inference that was made from the data or was this an explanation ascribed by the authors? As a general comment, I would be careful in the Results section to ground statements explicitly in the data, and reserve discussing implications or less obvious potential explanations for the Discussion section.

Thank you for this important comment. Our study used an Interpretative Phenomenological Analysis (IPA) approach, which focuses not only on describing participants' experiences but also on interpreting the underlying meaning within their specific context. Rather than presenting participant statements in isolation, we sought to understand the deeper influences shaping their perspectives, including how trust in services develops over time. We were not just interested in describing, but rather in understanding whats driving the surface level themes, the latent meaning.

We have clarified this on lines 255-260 and added a citation to support this interpretative approach.

16. Line 336: “even among initiators”- redundant, as you have to have initiated to be able to discontinue?

Agreed – we have removed this fragment.

17. Line 353-362: I thought this part was a bit more speculative, as the argument (although valid on its own) is entirely based on an uncertain explanation of PrEP discontinuation during pregnancy. Why was it considered likely by the authors that PrEP discontinuation was attributed to pregnancy here? I would specify or if insufficient data to back this, leave it out.

This information was given to us by the key informant who had a maternal role with the participant. She informed us that the underlying reason the participant stopped PrEP was because of her pregnancy, and shortly after this interview, the woman gave birth. We have reflected on your comment and have decided to leave out this information.

18. Line 431-432: there seems to be an editing issue with the sentence here.

We deleted this hanging fragment.

19. Line 523: I thought this was a bit of a difficult quote to follow as PrEP are also ARVs. Is this participant talking about HIV-positive colleagues here when talking about ARVs? Consider using a different quote if there are any that can make this point clearer?

We have removed a fragment of this quote to make it more clear. The participant is saying that women she brings into prostitution become HIV infected and start taking ARVs, but she remains negative because she is taking PREP. See lines 549-552 .

20. Line 575: As I was reading about this theme on flexibility, I had the reflection that considering PrEP only as an intervention relevant to the work setting (e.g. to be used when engaging in sex work activities) and not so much to sexual relationships with (stable) private partners might also carry a risk of generating a false feeling of safety, as HIV can also be acquired outside more “commercial” relationships. The authors may want to comment on this in the manuscript.

We have added this reflection to lines 807- 809: Care should be taken to balance messaging around PrEP’s flexibility, with education on HIV risk across different relationship types (client versus committed monogamous partnerships) to ensure informed decision-making.

We also add to the discussion lines 804-806 that Care should be taken to balance messaging around PrEP’s flexibility, with education on HIV risk across different relationship types (client versus committed monogamous partnerships) to ensure informed decision-making.

21. Line 600: is there a word missing (e.g. “even before LAI-PrEP rollout”)?

We have made this change, thanks for catching it.

22. Line 622: do the authors have any insights as to why consistent availability of LAI-PrEP was considered to be more of an issue or concern as compared to the consistent availability or oral PrEP? Was this rooted in experiences of stock disruptions for other injectable products (e.g. for family planning)?

Interestingly enough, both examples you have given are actually experienced in Zambia, and so, we have added both to Lines 653-654

23. Line 641: rather than infection by resistant strains, I think that suddenly stopping LAI-PrEP could foster the development of resistant strains due to suboptimal drug levels when infected with HIV.

Good point and thank you for the helpful wording to convey this idea more clearly. We have clarified this on line 672.

**Discussion**:
24. Generally well-written and balanced discussion. I would suggest including some more references. For instance, Line 770 (evidence for role-playing techniques or Q&A sessions?), Line 778-779 (evidence for peer-led group discussions?), and Line 825-829 (evidence for related interventions?).

Good suggestion – we have added nice papers with examples of interventions that have worked throughout Nigeria, South Africa and Zambia for sex workers.

25. Limitations: any limitations to the rapid analysis approach (cfr. what would a fully fletched thematic analysis have added to the analysis)? Do the authors consider any limitations regarding the self-reported nature of the data (e.g. related to social desirability or any other bias), or was this risk considered to be low in this context?

We acknowledge that rapid analysis differs from a fully developed thematic analysis, as it involves a more focused and expedited coding process. However, studies suggest that while rapid analysis may not capture all nuances, it reliably identifies key themes. To mitigate potential limitations, we contextualized our findings within existing literature and applied an Interpretative Phenomenological Analysis (IPA) approach, allowing us to move beyond surface-level descriptions to interpret participants' experiences in their specific contexts. See the following citations for more information:

1. Hamilton AB, Finley EP. Qualitative methods in implementation research: An introduction. Psychiatry Res. 2019 Oct;280:112516.

2. Qualitative Methods in Rapid Turn-Around Health Services Research [Internet]. 2013. (Health Services Research & Development Cyberseminar). Available from: https://www.hsrd.research.va.gov/for_researchers/cyber_seminars/archives/video_archive.cfm?SessionID=780

Regarding self-reported data and social desirability bias, we believe the risk was minimized in this study. The research team had established long-term relationships with the community through prior engagement efforts, fostering an environment of trust and openness. This rapport likely reduced pressure on participants to provide socially desirable responses.

We have added these reflections to lines 901-04.

**Reviewer #2:**

This manuscript describes a qualitative study of PrEP enablers and barriers in the setting of injectable cabotegravir rollout, including for female sex workers, in Zambia. Whereas the findings have largely been reported in previous research, the paper is a valuable addition to the literature, given the lack of literature on sex worker perceptions of long-acting cabotegravir in sub-Saharan Africa. The paper also serves as a helpful reminder that the same barriers encountered during oral PrEP rollout persist as long-acting PrEP is scaled up. Of interest is the perception that injectable PrEP is empowering because it can be used discreetly and circumvent the HIV-associated stigma associated with oral PrEP. A strength of the study is that community based participatory research methods were used to design and pilot-test the interview guide.

My overarching comment is that the “arguments” in the theme descriptions should primarily reflect the viewpoints, experiences, and voices of the interviewees (participants’ voices) rather than the researcher’s observations (analyst's voice, e.g., lines 353-362, 640-644), which are more appropriately placed in the discussion section.

Additionally, the results are pretty dense (15 themes spanning 14 pages), and it would be helpful if the authors distilled their key findings into a qualitative results summary that tells a coherent story for the reader's benefit. I have a few comments which the authors might find helpful.

Thank you for this important comment. Our study used an Interpretative Phenomenological Analysis (IPA) approach, which focuses not only on describing participants' experiences but also on interpreting the underlying meaning within their specific context. Rather than presenting participant statements in isolation, we sought to understand the deeper influences shaping their perspectives, including how trust in services develops over time. We were not just interested in describing, but rather in understanding whats driving the surface level themes, the latent meaning.

We have clarified this on lines 255-260 and added a citation to support this interpretative approach.

We appreciate the feedback on the structure of our results. However, our analysis is firmly grounded in the COM-B theoretical framework, and each of the 15 themes aligns with specific constructs and domains within this model. Given this theoretical orientation, reducing the number of themes would risk omitting critical aspects of the data that contribute to a comprehensive understanding of the factors influencing PrEP use.

Table 1 serves to summarize the qualitative results.

1) Interpretation: The description of themes should be directly supported by evidence from participant quotes that illustrate the lived experiences behind the theme. However, thematic descriptions are only sometimes backed up by evidence in illustrative quotes. For example, the elaboration in lines 690-696 is not supported by evidence from quotes and appears to reflect the researchers’ observations. Medical mistrust (lines 400, 415) is not evidenced by the quotes (lines 410-413, 420-423), which describe enacted interpersonal stigma in healthcare settings.

Thank you for this important comment. Our study used an Interpretative Phenomenological Analysis (IPA) approach, which focuses not only on describing participants' experiences but also on interpreting the underlying meaning within their specific context. Rather than presenting participant statements in isolation, we sought to understand the deeper influences shaping their perspectives, including how trust in services develops over time. We were not just interested in describing, but rather in understanding whats driving the surface level themes, the latent meaning.

We have clarified this on lines 255-260 and added a citation to support this interpretative approach. This is why the elaboration in lines 690-696 is discussed.

We have added clarification that the quotes are discribing discrimination from health care providers (also known as enacted stigma) on lines 439-440, but overall the quotes that have been listed are showing that women experience discrimination in the government clinics, and this does contribute to their lack of trust in the government health system. This is a barrier to them using those services, because multiple quotes show that they do not return for care.

2) Methods – Interviewer effect: A limitation of the study is that a single interviewer conducted all interviews, and there is potential for bias because one person's perspective could have influenced the questions asked, potentially skewing the results. This should be acknowledged in the Limitations.

We acknowledge the potential for interviewer bias as a limitation, given that a single researcher conducted all interviews. This was a pragmatic choice based on resource availability, as the study was conducted as part of a doctoral research project. However, this interviewer had extensive experience in HIV research and longstanding engagement with the sex work community, including work with community advisory boards on the parent study. Their established relationships likely fostered trust and open dialogue, helping to mitigate interviewer effects. Additionally, reflexivity was maintained throughout the research process to ensure that data interpretation remained grounded in participants' perspectives rather than the interviewer’s own assumptions.

This was added to lines 905-908.

3) The Results section is written in present and past tense. Using the past tense is customary since you describe findings derived from completed data collection and analysis.

We have gone through the entire Results section and standardized the tense to past tense. Thanks for this good advice. We feel that it reads much better now.

4) Results, Theme 3 (alcohol use is part of the lifestyle, but incompatible with daily pill adherence). The quotes provided support alcohol use as a coping strategy but do not provide direct evidence that alcohol use is incompatible with adherence. The third quote alludes to forgetting pill taking when drunk, but this is not the same as “incompatibility with daily pill adherence.”

Thank you for this comment. In HIV programs, daily pill adherence is defined as taking medication consistently at the prescribed frequency. This quote indicated that alcohol use led to forgetfulness and inconsistent dosing, making it difficult to adhere to the daily regimen. While alcohol use itself is not inherently incompatible with PrEP, participants’ experiences suggest that frequent drinking disrupted their ability to take PrEP reliably, which aligns with adherence challenges documented in other studies.

5) Results, lines 353-362: The authors provide an anecdote about a pregnant participant going into labour shortly after completing her in-depth interview. Since these observations about person-centered care are not directly derived from participant interviews, they are better placed in the Discussion.

This information was given to us by the key informant who had a maternal role with the participant. She informed us that the underlying reason the participant stopped PrEP was because of her pregnancy, and shortly after this interview, the woman gave birth. We have reflected on your comment and have decided to leave out this information.

6) **Discussion**:

The authors state that their study is the first to describe significant knowledge gaps and misconceptions about PrEP adherence and alcohol use as barriers. At least 18 studies on PrEP awareness and interest among people with unhealthy alcohol use were reviewed in a scoping review (AIDS Behav. 2020 Nov 20;25(6):1777–1789), including among young women in Kenya (AIDS Res Hum Retroviruses. 2016;32((Supplement 1)):384) and sex workers in Zambia (BMJ Public Health 2024;2:e000483).

Thank you for these citations – we have added them to our paper. We have also changed the language to be more conservative rather than boldly proclaiming we were the first study to discuss these topics.

7) Line 176: “Women are sometimes given the date for next PrEP refill…” Using the word “sometimes” makes it seem that appointment dates are optional.

We have removed the word ‘sometimes’ as the appointment dates are not optional.

8) Lines 202-203: “will be available in Zambia next year”. For specificity, perhaps replace “next year” with 2024.

Good point - we have made this substitution.

9) Lines 231-232: Incomplete sentence: “health education, particularly in encouraging the acceptance and use of PrEP among WESW.”

We have remove this sentence fragment which remained from multiple revisions.

10) Lines 583-584: The word “when” at the beginning of the sentence changes its meaning. Removing it implies the interviewee would stop taking PrEP after she found a suitor, which is probably the intended meaning.

We have changed this quote to reflect the intended meaning as you have pointed out, which is that the interviewee would stop PrEP after finding a husband.

11) Line 600: Add a comma after LAI-PrEP to improve sentence comprehension.

We have added “even before LAI-PrEP rolled out,”

12) Lines 640-644: The analyst’s voice should be in the Discussion section.

We have rewritten these lines to make it clear that the analyst is reflecting on the participants.

13) Line 722 should read, “Two women described this phenomenon.”

Good catch – we have made this edit.

14) Line 755: “Our findings on the barriers to oral PrEP initiation…” What about injectable PrEP, which was a key focus of the study?

We have revised this to include injectable PrEP. Thank you for catching this.

15) Lines 755-757 could be better referenced with other studies of PrEP misconceptions, e.g., AIDS Behav. 2021 Aug;25(8):2517-2532, Arch Sex Behav. 2021 May;50(4):1729-1742, Front Public Health. 2022 Jun 17;10:691729).

Thank you for these excellent references! We have included the ones relevant to the experiences of sex workers in our paper.

16) Line 842: What does “this may income brothel leadership” mean?

This was a typo which we have corrected to say “this may include brothel leadership.” Thank you for catching this.

6. PLOS authors have the option to publish the peer review history of their article ([what does this mean?](https://track.editorialmanager.com/CL0/https:%2F%2Fjournals.plos.org%2Fglobalpublichealth%2Fs%2Feditorial-and-peer-review-process%23loc-peer-review-history/1/010f0193c06a0562-70d520ea-1c6f-44a4-9e09-5d26128b3d22-000000/Pmj-U8xifeUDknF_aT8ZjRrSjVSAaialnvPMj23zEc4=189)). If published, this will include your full peer review and any attached files.


**Do you want your identity to be public for this peer review?** If you choose “no”, your identity will remain anonymous but your review may still be made public.


For information about this choice, including consent withdrawal, please see our [Privacy Policy](https://track.editorialmanager.com/CL0/https:%2F%2Fwww.plos.org%2Fprivacy-policy/1/010f0193c06a0562-70d520ea-1c6f-44a4-9e09-5d26128b3d22-000000/BCRaZ6P1sVWS7psz1SSeKyRs5YkBvcan8Vu4sMkDbF8=189).

Reviewer #1: **Yes:** Jef Vanhamel

Reviewer #2: No
